# Aging restricts the ability of mesenchymal stem cells to promote the generation of oligodendrocytes during remyelination

**DOI:** 10.1002/glia.23624

**Published:** 2019-04-30

**Authors:** Francisco J. Rivera, Alerie G. de la Fuente, Chao Zhao, Maria E. Silva, Ginez A. Gonzalez, Roman Wodnar, Martina Feichtner, Simona Lange, Oihana Errea, Eleni Priglinger, Anna O'Sullivan, Pasquale Romanelli, Janusz J. Jadasz, Gabriele Brachtl, Richard Greil, Herbert Tempfer, Andreas Traweger, Luis F. Bátiz, Patrick Küry, Sebastien Couillard‐Despres, Robin J. M. Franklin, Ludwig Aigner

**Affiliations:** ^1^ Laboratory of Stem Cells and Neuroregeneration, Institute of Anatomy, Histology and Pathology Faculty of Medicine, Universidad Austral de Chile Valdivia Chile; ^2^ Center for Interdisciplinary Studies on the Nervous System (CISNe) Universidad Austral de Chile Valdivia Chile; ^3^ Institute of Molecular Regenerative Medicine Paracelsus Medical University Salzburg Austria; ^4^ Spinal Cord Injury and Tissue Regeneration Center Salzburg (SCI‐TReCS) Paracelsus Medical University Salzburg Austria; ^5^ Wellcome‐MRC Cambridge Stem Cell Institute & Department of Clinical Neurosciences University of Cambridge Cambridge UK; ^6^ Institute of Pharmacy, Faculty of Sciences Universidad Austral de Chile Valdivia Chile; ^7^ Ludwig Boltzmann Institute for Experimental and Clinical Traumatology AUVA Research Center Linz/Vienna Austria; ^8^ Austrian Cluster for Tissue Regeneration Vienna Austria; ^9^ Institute of Experimental Neuroregeneration Paracelsus Medical University Salzburg Salzburg Austria; ^10^ Laboratory of Experimental Ophthalmology, Department of Ophthalmology University Hospital Düsseldorf, Heinrich‐Heine‐University Düsseldorf Germany; ^11^ Laboratory for Immunological and Molecular Cancer Research, 3rd Medical Department for Hematology, Medical Oncology, Hemostasiology, Infectious Diseases, and Rheumatology Federal Hospital of Salzburg and Paracelsus Medical University Salzburg Austria; ^12^ Experimental and Clinical Cell Therapy Institute Paracelsus Medical University Salzburg Austria; ^13^ Institute of Tendon and Bone Regeneration Paracelsus Medical University Salzburg Austria; ^14^ Centro de Investigación Biomédica (CIB), Facultad de Medicina Universidad de los Andes Santiago Chile; ^15^ Department of Neurology, Medical Faculty Heinrich‐Heine‐University Düsseldorf Germany

**Keywords:** aging, cell therapy, CNS stem and progenitor cells, mesenchymal stem cells, multiple sclerosis, oligodendrocytes, remyelination

## Abstract

Multiple sclerosis (MS) is a demyelinating disease of the central nervous system (CNS) that leads to severe neurological deficits. Due to their immunomodulatory and neuroprotective activities and their ability to promote the generation of oligodendrocytes, mesenchymal stem cells (MSCs) are currently being developed for autologous cell therapy in MS. As aging reduces the regenerative capacity of all tissues, it is of relevance to investigate whether MSCs retain their pro‐oligodendrogenic activity with increasing age. We demonstrate that MSCs derived from aged rats have a reduced capacity to induce oligodendrocyte differentiation of adult CNS stem/progenitor cells. Aging also abolished the ability of MSCs to enhance the generation of myelin‐like sheaths in demyelinated cerebellar slice cultures. Finally, in a rat model for CNS demyelination, aging suppressed the capability of systemically transplanted MSCs to boost oligodendrocyte progenitor cell (OPC) differentiation during remyelination. Thus, aging restricts the ability of MSCs to support the generation of oligodendrocytes and consequently inhibits their capacity to enhance the generation of myelin‐like sheaths. These findings may impact on the design of therapies using autologous MSCs in older MS patients.

## 1 INTRODUCTION

Multiple sclerosis (MS) is an autoimmune demyelinating disease of the central nervous system (CNS), commonly with onset in young adulthood and progression throughout life (Reich, Lucchinetti, & Calabresi, 2018). Therefore, the prevalence of MS is high in the aging population. In response to demyelination, CNS‐resident oligodendrocyte progenitor cells (OPCs) become activated, proliferate, and migrate to the lesion and differentiate into new oligodendrocytes, completing remyelination (Franklin & Ffrench‐Constant, 2017). While in MS and in other demyelinating diseases, remyelination occurs efficiently in young adults, with aging OPCs' differentiation and maturation into oligodendrocytes declines, becoming a limiting factor for spontaneous remyelination (Sim, Zhao, Penderis, & Franklin, 2002).

Mesenchymal stem cells (MSCs) are undifferentiated multipotent stromal cells able to generate osteoblasts, adipocytes as well as chondrocytes, and capable of modulating the microenvironment through paracrine signals (Minguell, Erices, & Conget, 2001). Therefore, MSCs are viewed as cells with many potential clinical applications, in particular as autologous cell therapies (for review see Ding, Shyu, & Lin [2011]). In the context of MS, MSCs have several potential benefits. They are immunomodulatory and can therefore be used to reduce the tissue damage resulting from acute inflammatory episodes that are typical for the early stages of the disease. Indeed, transplanted MSCs are immunomodulatory and neuroprotective promoting functional recovery in MS animal models (Bai et al., 2009, 2012) and apparently elicit beneficial effects in human MS patients (Connick et al., 2012).

MSCs’ potential role in myelin regeneration is still being explored. While MSCs do not directly transdifferentiate to myelin‐producing cells (Hunt et al., 2008), there is accumulating evidence suggesting that MSCs might beneficially influence remyelination by modulating endogenous progenitor activities. We have previously demonstrated that soluble factors derived from MSCs induce an oligodendrocyte fate on neural stem cells (NSCs; Rivera et al., 2006; Steffenhagen et al., 2012) and enhance OPC differentiation (Jadasz et al., 2013). Furthermore, cotransplantation of NSCs together with MSCs onto organotypic hippocampal slice cultures promoted oligodendrocyte differentiation of the transplanted NSCs (Rivera et al., 2009). Also, MSCs grafted into the fimbria of cuprizone‐induced demyelinated mice significantly activated OPCs and supported remyelination (Jaramillo‐Merchan et al., 2013). This capacity of MSCs to promote oligodendrogenesis is not restricted to rodents as soluble factors derived from human MSCs induce oligodendrocyte differentiation in human induced pluripotent stem cell‐derived NSCs (Jadasz et al., 2018). In summary, MSC transplantation might be developed into a potentially powerful MS therapy (Connick et al., 2012; Freedman et al., 2010; Jadasz, Aigner, Rivera, & Kury, 2012). Here we ask whether MSCs retain their capacity to enhance the generation of new oligodendrocytes with aging.

## 2 MATERIALS AND METHODS

### 2.1 Animal subjects

Two‐month‐old and 17–20 months old male and female Fischer 344 rats (Charles River Deutschland GmbH, Germany) were used as young and old donors, respectively, for MSC cultures. Demyelinating surgeries were performed in 12‐month‐old male Fisher 344 rats (Harlan, UK) according to Home Office regulations. P0–P2 Sprague–Dawley rats (Internal Breeding Stock, University of Cambridge) were used for OPC cultures. Animal experiments within this research have been regulated under the Animals (Scientific Procedures) Act 1986 Amendment Regulations 2012 following ethical review by the University of Cambridge Animal Welfare and Ethical Review Body (AWERB) in accordance with UK Home Office regulations (Project License: 70/7715).

### 2.2 MSC cultures

MSC cultures from young and old rats were performed as previously described (Rivera et al., 2008). Briefly, bone marrow (BM) plugs were harvested from femurs and tibias of young (y; 2‐month‐old) and old (o; 17‐ to 20‐month‐old) male and female Fisher‐344 rats (Charles River Deutschland GmbH, Germany). Plugs were mechanically dissociated in αMEM (Gibco Invitrogen, Karlsruhe, Germany) and recovered by centrifugation. Cell pellets were resuspended in αMEM containing 10% FBS (PAA, Austria) and 1% Penicillin/Streptomycin (PAN Biotech GmbH, Aiden Bach, Germany) (αMEM‐10% FBS) and seeded at 1 × 10^6^ cells/cm^2^. After 3 days, medium was changed, and nonadherent cells were removed. Adherent cells were incubated in fresh αMEM‐10% FBS until a confluent layer of cells was achieved. Cells were trypsinized using 0.25% Trypsin (Gibco Invitrogen, Karlsruhe, Germany) and seeded in αMEM‐10% FBS at 8,000 cells/cm^2^. After 3–5 days of culture, the resulting monolayer of cells, hereafter named rat bone marrow‐derived MSCs, was trypsinized and further cultured for experiments or frozen for later use. As demonstrated in our previous work, this cell culture preparation is highly enriched in multipotent MSCs with no more than 4.5% of hematopoietic contamination (Rivera et al., 2006). We named yMSCs when cells were isolated from young rats (2‐month‐old) and oMSCs (17‐ to 20‐month‐old) when were isolated from old animals.

### 2.3 NSC cultures

Tripotent NSCs derived from rat hippocampus were generated as described (Wachs et al., 2003). Briefly, hippocampi from 2‐month‐old male and female Fisher‐344 rats (Charles River Deutschland GmbH, Germany) were aseptically removed and dissociated. Cells were resuspended in Neurobasal‐A medium (NB‐A; Gibco BRL, Germany) supplemented with B27 (Gibco BRL, Germany), 2 mM l‐glutamine (PAN, Germany), 100 U/mL penicillin/0.1 mg/L streptomycin (PAN, Germany), hereafter referred to as NB/B27. For maintenance and expansion of the cultures, the NB/B27 was further supplemented with 2 mg/mL heparin (Sigma, Germany), 20 ng/mL FGF‐2 (R&D Systems, Germany), and 20 ng/mL EGF (R&D Systems, Germany). As previously described (Rivera et al., 2006), immediately after plating of dissociated neurospheres in NB/B27‐5% FBS, around 90% of the NSCs expressed the neural stem and progenitors markers such as nestin and A2B5. Cultures were maintained at 37°C in a humidified incubator with 5% CO_2_. Neurosphere cultures from passage number 2–6 were used throughout this study and termed NSCs.

### 2.4 OPCs cultures

OPCs were obtained from P0–P2 Sprague–Dawley rats (Internal Breeding Stock, University of Cambridge). Briefly, purified OPCs were obtained following the protocol of McCarthy and De Vellis. Cells from the cortex and hippocampus were dissociated and cultured in the presence of DMEM (Gibco, Carlsbad, CA) containing 1% P/S (Sigma Aldrich, Buchs, Switzerland) and 10% fetal bovine serum (FBS, Biosera, Boussens, France) on poly‐d‐lysine coated flasks. After 10–12 days of mix glial cultures OPCs were mechanically dissociated from other glial cell types (McCarthy & de Vellis, 1980). OPCs were isolated by shaking off loosely adherent cells from the astrocyte adherent cell monolayer. The supernatant (enriched in OPCs) was collected and microglial cells were removed by incubation in plastic Corning petri dishes (BD, Oxford, UK) 15 min at 37°C. As previously described, this procedure results in a relatively pure population of OPCs (de la Fuente et al., 2015). Indeed, we determined that 92 ± 4% of total cells were Olig2‐expressing undifferentiated OPCs. These cells are positive for the oligodendroglial lineage marker Olig2 and negative for the expression of mature oligodendrocyte markers, such as MBP and CNPase. OPCs were resuspended and plated in 13 mm poly‐d‐lysine (5 μg/mL) precoated coverslips (Thermo Scientific, Waltham, MA) in serum‐free Sato media (DMEM supplemented with 0.3 mg/mL BSA fraction V [Sigma Aldrich], 0.1 mg/mL progesterone, 1.61 mg/mL putrescine, 25 μg/mL sodium selenite, 1% P/S 50 μg/mL of holo‐Transferrin [Sigma Aldrich], and 5 μg/mL of insulin [Gibco]). OPCs were cultured in Sato media supplemented with 10 ng/mL of PDGF‐AA and 10 ng/mL bFGF (Peprotech, Rocky Hill, NJ) to keep the cells in the proliferative stage.

### 2.5 Organotypic cerebellar slices cultures

Demyelinating rat cerebellar slice cultures were prepared as previously described (Birgbauer, Rao, & Webb, 2004). Briefly, P9 rat cerebellum was isolated and 300 μm sections were obtained using McIlwain tissue chopper (McIlwain, San Antonio, TX). Slices were then incubated at 37°C with 5% CO_2_ in organotypic slice medium (50% BME [Gibco], 25% heat‐inactivated horse serum [Gibco], 25% HBSS [BME, Gibco], 5 mg/mL glucose, 1× glutamax [Gibco], and 1× Mycozap‐PR plus [Lonza]). After a week in culture, slices were demyelinated with 0.5 mg/mL lysolecithin for 16 hr and then incubated in lysolecithin‐free medium to allow remyelination.

### 2.6 Growth curve and cell number determination

yMSCs and oMSCs from different passage number were plated at 5,000 cells per cm^2^ and incubated in MSCs proliferation medium (αMEM‐10% FBS). The correct number of seeded cells was immediately confirmed using CASY‐Cell Counter (Roche, Vienna Austria). Cells were incubated up to 1 week in αMEM‐10% FBS. After 1, 3, 5, and 7 days incubation cells were detached with 0.25% Trypsin (Gibco BRL, Karlsruhe), collected in falcon tubes, centrifuged at 600*g* for 10 min and the cell pellet was resuspended in 1 mL αMEM‐10% FBS. About 250 μL of each cell suspension was dissolved in CASYTON (Roche, Vienna Austria) and cell number was determined with CASY‐Cell Counter (Roche, Vienna Austria). Measurements with CASY‐Cell Counter were performed using the following settings: Dilution: 2.000e +00, Capillary: 150 μm, *X*‐axis: 30 μm, sample volume: 400 μL, cycles: 3, evaluation cursor: 9.00–30.00 μm, normal cursor 15–30.00 μm.

### 2.7 MSC‐conditioned media preparation

For experiments performed in NSCs, MSC‐conditioned medium (MSC‐CM) was prepared as described (Rivera et al., 2006). Briefly, yMSCs or oMSCs were plated at 12,000 cells/cm^2^ and incubated in MSC proliferation medium (αMEM‐10% FBS). For experiments performed in OPCs, yMSCs, or oMSCs were incubated with serum free Sato media (DMEM supplemented with 0.3 mg/mL BSA fraction V [Sigma Aldrich], 0.1 mg/mL progesterone, 1.61 mg/mL putrescine, 25 μg/mL sodium selenite, 1% P/S 50 μg/mL of holo‐Transferrin [Sigma Aldrich], and 5 μg/mL of insulin [Gibco]) without PDGF‐AA and bFGF. For experiments performed in demyelinated cerebellar slices, yMSCs, or oMSCs were incubated with organotypic slice culture medium (50% BME [Gibco], 25% heat inactivated horse serum [Gibco], 25% HBSS [BME, Gibco], 5 mg/mL glucose, 1× glutamax [Gibco] and 1× Mycozap‐PR plus [Lonza]). Conditioned media was collected after 3 days *in vitro* and filtered through a 0.22 μm‐pore filter.

### 2.8 Bioluminescence assays

Bioluminescence assays were performed using noncommercial dual luciferase enzyme assay system. In this system, NSCs were cotransfected with (a) plasmid containing MBP promoter driving the expression of firefly luciferase (pMBP‐Luci); (b) control vector that contains the cytomegalovirus (CMV) promoter driving the expression of Renilla luciferase (Promega, Mannheim, Germany). The two different luciferases use different specific substrates and therefore, the MBP regulatory region driven firefly luciferase activity was distinguished from Renilla luciferase. Transfected NSCs were seeded in Poly‐l‐Ornithine/Laminin‐coated dishes in white‐96‐well plates and incubated either under control condition (αMEM‐10% FBS) or in different MSC‐CM dilutions. After 3 days NSCs were exposed to lysis solution that contained 25 mM Tris‐phosphate pH 7.8, 2 mM DTT, 1% Triton X‐100 (Sigma‐Aldrich, Taufkirchen, Germany), 2 mM EDTA, 10% Glycerol (Merck, Darmstadt, Germany). To analyze the firefly luciferase activity cell lysate was treated with the following buffer: 25 mM glycylglycine (Acros, Geel, Belgium) 15 mM K_x_PO_4_ pH 8.0, 4 mM EGTA, 15 mM MgSO_4_ (Merck, Darmstadt, Germany), 2 mM ATP, 1 mM DTT, 0.1 mM Coenzyme A, 75 mM luciferin (Sigma‐Aldrich, Taufkirchen, Germany). To analyze the Renila luciferase activity cell lysate was treated with the following buffer: 1.1 M NaCl (VWR, Vienna, Austria), 2.2 mM Na_2_EDTA, 0.22 M K_x_PO_4_ pH 5.1 (Merck, Darmstadt, Germany), 0.44 mg/mL BSA (Biomol, Hamburg, Germany), 1.3 mM NaN3 (Sigma‐Aldrich, Taufkirchen, Germany), 1.43 mM coelenterazine (P.J.K., Kleinblittersdorf, Germany). Bioluminescence was measured by Luminometer (Tristar LB 941, Berthold Technologies, HVD, Austria) and luciferase activity was determined. In all conditions, firefly luciferase activity was normalized against the Renilla luciferase activity.

### 2.9 NSCs, OPCs, and demyelinated cerebellar slices stimulation with yMSC‐CM and oMSC‐CM

NSCs were treated with yMSC‐CM and oMSC‐CM as previously described (Rivera et al., 2006). Briefly, NSCs were plated overnight onto poly‐ornithine (250 μg/mL) and laminin (5 μg/mL)‐coated glass coverslips at a density of 12,000 cells/cm^2^ in αMEM‐10% FBS. Next, media was replaced and cells were incubated either with yMSC‐CM or oMSC‐CM. NSCs were alternatively incubated in αMEM‐10% FBS as control condition. Media was changed every third day of incubation. After 1 week of incubation, or alternatively after 3 days in the luciferase experiments, cells were fixed for 30 min with phosphate‐buffered 4% paraformaldehyde (Sigma‐Aldrich, Taufkirchen, Germany) and processed either for immunofluorescence, or for bioluminescence analysis, respectively.

OPCs were treated with yMSC‐CM and oMSC‐CM. OPCs were plated in 13 mm poly‐d‐lysine precoated coverslips (Thermo Scientific) at a density of 30,000 cells per cm^2^. Cells were incubated for 3 days in Sato‐based serum‐free media supplemented daily with 10 ng/mL of PDGF‐AA and 10 ng/mL bFGF to keep the cells in the proliferative stage. Next, media was switched to Sato‐based yMSC‐CM or oMSC‐CM in the absence of growth factors. OPCs were alternatively incubated in Sato media without growth factors as a control condition. Media was replaced every 2 days and OPCs were fixed with phosphate‐buffered 4% paraformaldehyde (pH 7.4) at 2 and 4 days of incubation and cells were processed for immunofluorescence.

After exposure to lysolecithin, demyelinated cerebellar slices were incubated with organotypic slice medium‐based yMSC‐CM or oMSC‐CM. Unconditioned organotypic slice medium was used as control. Treatment was replaced every other day and slices were collected at 2 and 6 days *in vitro* (DIV) post demyelination and fixed with 4% PFA for 1 hr. Then slices were stored in phosphate‐buffered saline (PBS) 1× at −20°C for immunostaining.

### 2.10 Immunocytochemistry and analysis

Fixed NSCs were washed in TBS (0.15 M NaCl, 0.1 M Tris–HCl, pH 7.5), then blocked with solution composed of TBS, 0.1% Triton‐X100 (only for intracellular antigens), 1% bovine serum albumin (BSA), and 0.2% Teleostean gelatin (Sigma, Germany; fish gelatin buffer [FGB]). The same solution was used during the incubations with antibodies. Primary antibodies were applied overnight at 4°C. Fluorochrome‐conjugated species‐specific secondary antibodies were used for immunodetection. The following antibodies and final dilutions were used. Primary antibodies: rabbit anti‐GFAP 1:1000 (Dako, Denmark), mouse anti‐Myelin Basic Protein (MBP) 1:750 (SMI‐94, Covance, Anopoli Biomedical Systems, Eichgraben, Austria), and rabbit. Secondary antibodies: donkey anti‐mouse, rabbit conjugated with Alexa Fluor 488, Alexa 568 1:1000 (Molecular Probes, Eugene, OR). Nuclear counterstaining was performed with 4′,6′‐diamidino‐2‐phenylindole dihydrochloride hydrate at 0.25 μg/μl (DAPI; Sigma, Germany). Specimens were mounted on microscope slides using the Prolong Antifade kit (Molecular Probes). Epifluorescence observation and photo‐documentation were realized using an Olympus IX81 microscope (Olympus, Germany) equipped with Hamamatsu digital camera and Volocity software (Perkin Elmer, Germany). For each culture condition, 5–10 randomly selected observation fields, containing in total 500–1,000 cells, were photographed for cell fate analysis. Expression frequency of selected cell type markers was determined for every condition in four independent experiments.

Fixed OPCs were washed in PBS before blocking. Next, cells were blocked with 5% normal donkey serum (Sigma, UK) and 0.1% Triton (Sigma, UK) in PBS for 1 hr at room temperature. The same solution was used during the incubation with antibodies. Primary antibodies were applied overnight at 4°C (rat anti‐Myelin Basic Protein (MBP) 1:500 (Serotec), mouse anti‐CNPase 1:400 (Abcam, UK) and goat anti‐Olig2 1:200 (Abcam, UK). After three washes with PBS fluorochrome‐conjugated species‐specific secondary antibodies (488 anti‐Goat 1:300 and Alexa fluor 568 anti‐rat 1:500 or Alexa Fluor 647 anti‐mouse 1:500 (Life Technologies, UK) were used for 2 hr at room temperature to detect primary antibodies. Nuclear counterstaining was performed with Hoechst 2 μg/mL (Sigma Aldrich). Epifluorescence observation and photo‐documentation were realized using an Olympus IX81 microscope (Olympus, Germany) equipped with Hamamatsu digital camera and Volocity software (Perkin Elmer, Germany). Percentage of cells expressing Olig2 that also expressed the other mature OPC markers was determined in at least three independent experiments.

### 2.11 Focal demyelination lesions

Male Fischer 344 rats aged 12 months were used to induce bilateral focal demyelination in the caudal cerebellar peduncle (CCP) in compliance with UK Home Office regulations (Project License: 70/7715, formerly 80/2228). Animals were anesthetized using a combination of 2% isoflurane in oxygen (as carrier gas) and a subcutaneous injection of buprenorphine hydrochloride 0.03 mg/kg (Vetergesic, Animalcare Ltd, Hull, UK). The procedure consisted on bilateral stereotactical injections of 4 μL of 0.01% ethidium bromide (v/v) into the CCP, −11.6 mm caudal, ±2.9 mm lateral, and −8.3 mm ventral from bregma (Woodruff & Franklin, 1999).

### 2.12 MSCs stable transfection with mCherry

PiggyBac transposon‐based strategy was used for MSCs stable transfection with mCherry (Yusa, Zhou, Li, Bradley, & Craig, 2011). The coding region of mCherry was amplified by PCR and cloned into the XbaI/EcoRI sites of the backbone PiggyBac vector PB‐CMV‐MCS‐EF1‐Puro (System Biosciences; Cat. No. PB5105B‐1). For further genomic integration, the following vector was used: pCMV‐hyPBase (provided by the Wellcome Trust Sanger Institute). MSCs obtained from 2 and 20 months old male rats were transfected with PB‐CMV‐mCherry‐EF1‐Puro and pCMV‐hyPBase plasmids. Amaxa® Basic Nucleofector® Kit for Primary Mammalian Smooth Muscle Cells (Lonza) and the program U‐023 were used to transfect 5 × 10^6^ cells with 4 μg of PB‐CMV‐mCherry‐EF1‐Puro and 1 μg of pCMV‐hyPBase. This methodology allowed approximately 40% of transfection efficiency. For further selection, transfected cells were incubated in the presence of 2 μg/mL puromycin (Sigma) for at least 1 week, leading to a highly pure mCherry‐expressing MSCs culture.

### 2.13 MSCs transplantation into demyelinated rats

mCherry‐expressing MSCs were intravenously transplanted into rats after CCP demyelination. To reduce the probability of transplant rejection, we used male Fisher 344 (inbred strain) rats as MSCs donors and transplanted host. Previous to transplantation, cultured mCherry‐expressing MSCs were collected in PBS at a suspension of 2.0 × 10^6^ cells/ml with a survival rate of 80–98%. Three daily doses of 1.5–2.0 × 10^6^ mCherry‐expressing MSCs obtained from young and old donors were administrated through the tail vein of previously demyelinated rats at 1, 2, and 3 days post‐lesion (dpl) induction. Tail vein infusion of PBS (1 mL) was used as vehicle control group. About 4–5 animals were considered per group. Animals were then perfused/fixed with 4% PFA at 21 dpl. Fixed brains were kept in 20% sucrose and embedded in optimal cutting temperature compound (Taab). Cryosections were obtained using a cryostat (Bright Instruments, Luton, Bedfordshire) at 12 μm thickness and then frozen at −80°C until further processing.

### 2.14 Immunohistochemistry

Fixed cerebellar slices were washed in PBS and blocked for 1 hr with 10% NGS 0.5% Triton X‐100 in agitation and incubated overnight with antibodies for MBP (1:500, Serotec, Kidlington, UK) and NFH (1:500, Abcam, Cambridge, UK) in blocking solution. Slices were washed three times with PBS and 0.1% Triton X‐100. Slices were incubated with fluorochrome‐conjugated secondary antibodies (AF488, AF568, 1:300, Invitrogen) for 2 hr at 20–26°C. The secondary antibodies were removed washing with PBS 0.1% Triton X‐100 and then Hoechst (1 μg/mL final concentration, Sigma) was used for nuclei staining. Slices were mounted with Fluoromount G onto poly‐d‐lysine slides, covered with a 20‐mm coverslip (VWR, Radnor, PA) and prepared for confocal microscopy visualization and documentation.

Twelve micrometers sections were dried for 1 hr at RT and washed with PBS. Only for Olig2/APC stainings, antigen retrieval was performed by incubating the sections for 10 min at RT with preboiled 1× citrate buffer, pH 6.0 and antigen retriever (Dako). No antigen retrieval was performed for mCherry stainings. Then, slides were incubated for 2 hr at RT with blocking solution (5% normal donkey serum and 0.1% Triton X‐100). After blocking, slices were incubated with primary antibodies overnight at 4°C, washed and incubated with secondary antibodies for 2 hr at RT in blocking solution. Nuclei were stained with 1 μg/mL Hoechst for 5 min, and slides were mounted after washes with Fluoromount G. Primary antibodies: rabbit anti‐Olig2 antibodies (1:200; R&D Systems), mouse anti‐APC (1:200; EMD Millipore), rabbit anti‐Ki67 (1:500; Abcam), mouse anti‐Nkx2.2 (1:100; Developmental Studies Hybridoma Bank), goat anti‐Iba1 (1:500; Abcam), rabbit anti‐mannose receptor (MR) (1:200; Abcam), rabbit anti‐Periaxin (1:1000; from Peter Brophy, Centre for Neuroscience Research, University of Edinburgh, UK), and rabbit anti‐mCherry (1:200; Abcam). Fluorochrome‐conjugated secondary antibodies: Donkey AF488 anti‐rabbit (1:500; Invitrogen), Donkey AF488 anti‐goat (1:500; Invitrogen), Donkey AF568 anti‐rabbit (1:500; Invitrogen), and Donkey AF647 anti‐mouse (1:500; Invitrogen).

### 2.15 Histological analysis and quantification

For the quantification and analysis of slices derived from the *ex vivo* organotypic culture, four independent slices were analyzed per condition and per experiment. Five images of randomly chosen areas of each slice were imaged using Leica SP5 confocal microscope 40× objective (NA 1.25) with 1.5 digital zoom, 1024 × 1024 resolution and 1.75–2 μm thickness stacks. Slices derived from the same animal were counted as *n* = 1 and the different biological experiments were counted as independent experiments (individual *n*). For analysis, a macro was created in Image J, which measured the MBP‐NFH colocalization area and the total NFH area.

For *in vivo* experiments, CCP lesions were recognized by the presence of increased cellularity denoted by Hoechst staining. For the quantification and analysis of the CCP lesion images, the whole lesion of three sections was imaged per animal with the 20× objective (NA 0.70), 1,024 × 1,024 resolution, and 1.5–2‐μm stacks. This was performed for each CCP lesion (left and right) per animal. Using ImageJ software, the lesion area was delineated based on Hoechst channel as mentioned previously and measured. Then the number of different cell types OPCs (Olig2+/APC−), proliferating OPCs (Nkx2.2+/Ki67+), mature oligodendrocytes (Olig2+/APC+), and Schwann cells (Periaxin+) within the lesion was counted manually with the cell counter plugin. The number of macrophages/microglia cells (Iba1+ and MR+) was quantified using ImageJ software by applying the following constant parameters of brightness/contrast, threshold, particle size, and circularity, respectively: 0/255, 75/255, 20–350, 0–40 (for Iba1) and 0/185, 45/255, 60–350, 0.05–40 (for MR). All the images were acquired at room temperature.

### 2.16 Oil red‐O staining

Sections from control and treatment group were left to dry at room temperature and then placed in a Coplin jar containing propylene glycol (100%) for 5 min. Prewarmed Oil Red‐O solution (0.5% in propylene glycol, Sigma‐Aldrich) was stained at 65°C for 10 min. Following incubation for 5 min with differentiation solution (85% propylene glycol), slides were rinsed twice with water and then mounted using a jelly‐based mounting media. Representative images of Oil Red‐O stained lesions were digitized, and, using ImageJ software, the intensity of the lesion area was measured from the red channel. Data are given as mean ± *SEM* and statistically analyzed. Three sections per marker per animal were analyzed, and a minimum of four animals were used.

### 2.17 Statistical analysis

Data are presented as means ± *SD* and statistical analysis was performed using PRISM4 (GraphPad, San Diego, CA) and SPSS (IBM, Headquarters). *p* values of <0.05 were considered to be significant acquired by parametric one‐way ANOVA and Tukey post hoc. For MSCs, growth curve and MSC‐CM dose‐dependent experiments, two‐way ANOVA and Bonferroni post hoc were performed. For experiments carried out in organotypic slices, two‐way ANOVA and Bonferroni post hoc were performed. For *in vivo* remyelination studies, 4–5 animals were considered per group and one‐way ANOVA/Tukey post hoc were performed. If not mentioned otherwise, experiments were performed in triplicates.

## 3 RESULTS

### 3.1 Aging decreases the capacity of MSCs to promote the generation of oligodendrocytes *in vitro*

**Figure 1:**
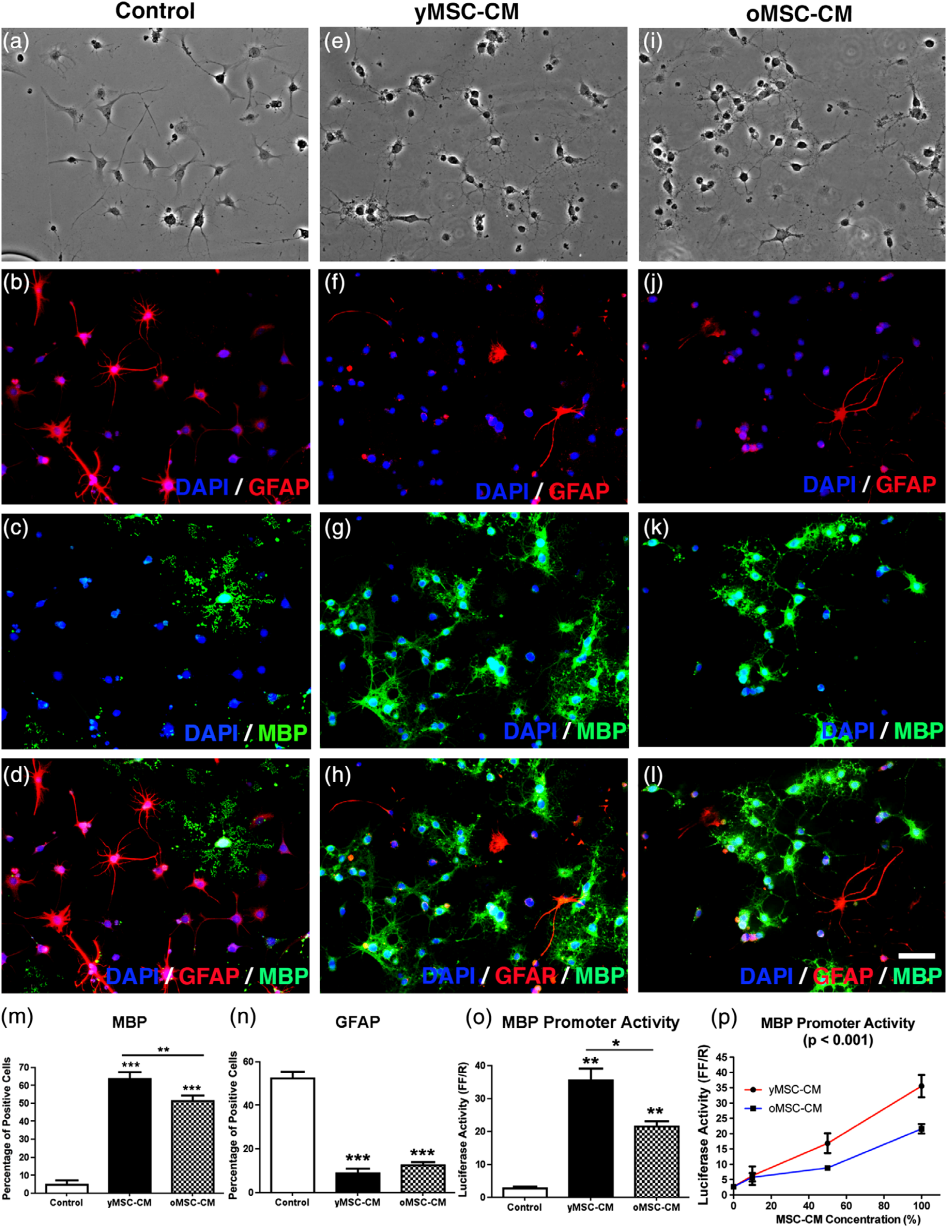
Aged MSCs display a reduced ability to promote the generation of oligodendrocytes from NSCs. NSCs were incubated in control medium (αMEM‐10%FBS, a–d) and MSC‐CM derived from young MSCs (yMSC‐CM, e–h) or old MSCs (oMSC‐CM, i–l) for 1 week. Phase contrast images show NSCs morphology after the different treatments (a, e, i). Fluorescent images indicate NSCs expression of GFAP (b, f, j), MBP (c, g, k), and the merge (d, h, l). DAPI nuclear staining is shown in all fluorescent images. (a–l) Scale bar = 50 μm. Quantitative analysis shows the percentage of MBP‐expressing cells (m) and GFAP‐expressing cells (n) for all conditions tested. Note that although both yMSC‐CM and oMSC‐CM induce an increase in the proportion of MBP‐expressing cells and a decrease in GFAP‐expressing cells with respect to control. However, yMSC‐CM displays a higher effect on MBP‐positive cells than oMSC‐CM. Values are displayed as mean ± *SD*. Experiment was performed in tetraplicate. One‐way ANOVA and the Tukey post hoc test were used for statistical analysis. **p < .01; *** *p* < .001. NSCs were transfected with MBP promoter‐Luci vector and incubated for 3 days under control medium, yMSC‐CM and oMSC‐CM. Cells were also exposed for 3 days to different MSC‐CM dilutions. Quantification of bioluminescence data shows MBP promoter activation (as luciferase activity) in NSCs either treated in the conditions previously mentioned (o) or treated with increasing doses of yMSC‐CM and oMSC‐CM (p). Note that although both yMSC‐CM and oMSC‐CM induce an increase of the MBP promoter activity with respect to control, yMSC‐CM displays a higher effect than oMSC‐CM. Values are displayed as mean ± SD. Experiment was performed in triplicate. One‐way ANOVA and the Tukey post hoc test as well as two‐way ANOVA (p) were used for statistical analysis. *p < .05; **p < .01 (o) and *p* values given in graph title indicate a significant difference between MSC‐CM ages (p)

Soluble factors derived from yMSCs promote oligodendrocyte fate decision and differentiation at the expense of astrogenesis in tripotent NSCs (Rivera et al., 2006). To determine whether MSC aging affects their pro‐oligodendrogenic activity, NSCs were incubated with conditioned media derived from yMSCs (yMSC‐CM) as well as from oMSCs (oMSC‐CM) and the oligodendrogenic effect was evaluated. NSCs under control conditions showed a typical flat and star‐like astrocytic morphology (Figure 1a), while cells incubated in yMSC‐CM and oMSC‐CM displayed a multipolar oligodendroglial morphology with secondary branches (Figure 1e,i). Most NSCs incubated under control conditions differentiated into glial fibrillary acidic protein (GFAP)‐expressing astrocytes (Figure 1b–d), whereas the majority of NSCs incubated in yMSC‐CM as well as oMSC‐CM give rise to myelin basic protein (MBP)‐expressing oligodendrocytes (Figure 1f–h and j–l, respectively). Both yMSC‐CM and oMSC‐CM decreased the proportion of GFAP‐expressing astrocytes compared to the control condition to a similar extent (Figure 1n). Thus, regardless of donor age, soluble factors derived from MSCs are able to induce an oligodendrocyte fate at the expense of astrogenesis. However, although both yMSC‐CM and oMSC‐CM increased the proportion of MBP‐expressing oligodendrocytes compared to the control, yMSC‐CM‐induced a higher percentage of MBP‐positive cells than oMSC‐CM in NSCs (Figure 1m). Bioluminescence assays were used to confirm these findings. Although both yMSC‐CM and oMSC‐CM‐induced an increase in MBP promoter activity compared to the control condition in a MSC‐CM concentration‐dependent manner (upon exposure to different MSC‐CM dilutions), NSCs incubated with yMSC‐CM displayed higher levels of luciferase activity compared to cells incubated in the presence of oMSC‐CM (Figure 1o,p). Since long‐term *in vitro* expansion alters the MSCs' growth rate ( Figure 1a,b) as well as inducing senescence in many cell types (Baxter et al., 2004), we evaluated whether MSC long term culture expansion might affect the oligodendrogenic capacity of MSCs. Thus, NSCs were exposed to conditioned medium obtained from MSCs of increasing passage number and MBP promoter activity was assessed by bioluminescence. Regardless of MSCs' donor age, long‐term *in vitro* expansion did not influence the MSCs derived oligodendrogenic activity, as MSC‐CM harvested from cells with high and low passage number similarly induced MBP promoter activity (Supporting Information Figure S1c,d). Overall, these results indicate that aging decreases the capability of MSCs to promote the generation of oligodendrocytes from NSCs.

**Figure 2:**
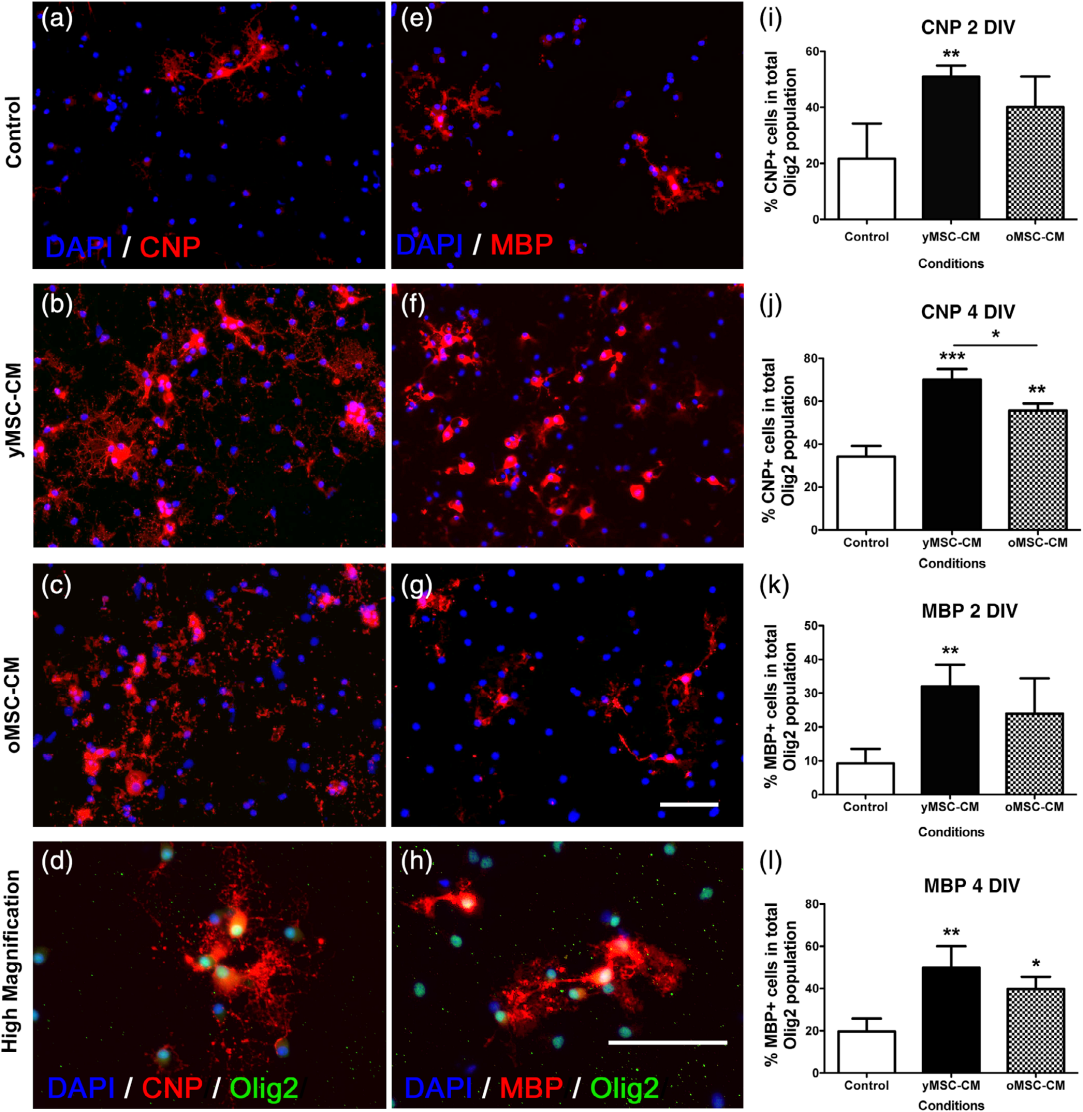
Aged MSCs display a reduced ability to promote OPC differentiation. Neonatal OPCs were incubated in control serum free Sato media (a and e) and yMSC (b and f) and oMSC (c and g) conditioned serum‐free Sato media for 2 and 4 days in vitro (DIV). Fluorescent images indicate the expression of differentiation CNP (red) and mature oligodendrocyte MBP (red) markers together with the pan oligodendrocyte lineage marker Olig2 (green). DAPI nuclear staining is shown in all fluorescent images. Quantitative analysis shows the percentage of CNP‐Olig2 (i and j) or MBP‐Olig2 (k and l) double‐positive cells within the Olig2 expressing cells at 2 and 4 days of incubation. Scale bars = 50 μm. Note that both, yMSC‐CM and oMSC increase OPC differentiation when compared to control. However, oMSC‐CM displays a delayed and less effective activity in OPCs as is only evident at 4 days of incubation and not so pronounced when compare to yMSC‐CM. Values are displayed as mean ± *SD*. Experiments were done in triplicate. Statistics were performed by one‐way ANOVA and the Tukey post hoc test. *p < .05; **p < .01; ***p < .001

As OPCs represent the major cellular source for remyelinating oligodendrocytes in the adult CNS (Zawadzka et al., 2010), we further asked whether young and old MSC were able to enhance OPC differentiation. Indeed, we have previously shown that MSCs‐derived soluble factors enhance OPC differentiation (Jadasz et al., 2013). Thus, OPCs were incubated for 2 and 4 days in the presence of yMSC‐CM or oMSC‐CM and differentiation was evaluated (Figure 2a–h). Sato based OPC media without growth factors was used as a control condition. Overall, independent of MSC donor's age, soluble factors derived from MSCs increased the proportion of Olig2‐expressing OPCs that differentiate into CNPase‐ and MBP‐expressing oligodendrocytes when compared to control media (Figure 2i–l). However, the yMSC‐CM pro‐oligodendrogenic effect on OPCs was more efficient compared to exposure to oMSC‐CM. When comparing with control media, 2 days of incubation with yMSC‐CM was sufficient to induce an increase in the generation of CNP‐ and MBP‐expressing oligodendrocytes, while this effect was only evident after 4 days of exposure to oMSC‐CM (Figure 2i–l). Moreover, the final proportion of CNP‐expressing mature oligodendrocytes was higher when OPCs were incubated with yMSC‐CM compared to oMSC‐CM (Figure 2j). yMSC‐CM might also promote proliferation and/or survival in OPCs, thus, indirectly increasing the proportion of mature oligodendrocytes rather than directly inducing OPC differentiation. To assess this possibility, we determined whether MSC‐CM influences the percentage of Ki67+ and Caspase3+ cells during OPC differentiation. No difference between yMSC‐CM and control conditions was observed (Supporting Information Figure S2a,b), indicating that yMSC‐CM enhances the generation of oligodendrocytes by promoting OPC differentiation rather than interfering with cell proliferation or survival. Due to the lack of effect of yMSC‐CM on OPC proliferation and survival, is very unlikely that MSC‐CM obtained from old donors exert this effect. Therefore, oMSC‐CM has a lower capacity to induce OPC differentiation compared to MSC‐CM derived from young donors.

In summary, both young and aged MSCs can promote the generation of oligodendrocytes, although aged MSCs do so less successfully.

### 3.2 Aging suppresses the ability of MSC‐CM to enhance *ex vivo* generation of myelin‐like sheaths

**Figure 3:**
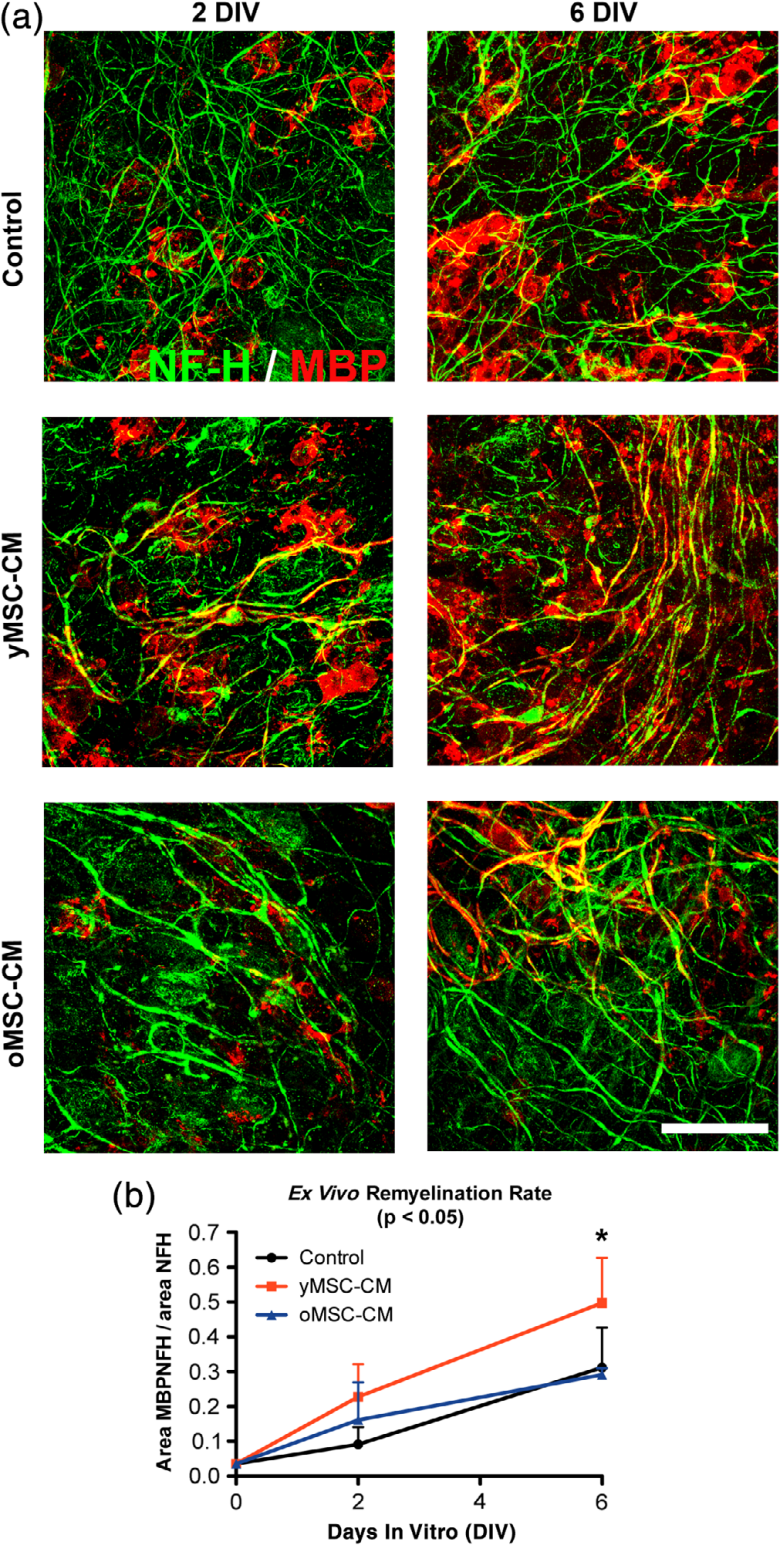
Soluble factors derived from yMSCs but not from oMSCs enhance myelin‐like sheath formation *ex vivo*. Cerebellar slices from P9 rats were cultured *ex vivo* and exposed for 16 hr to lysolecithin to induce demyelination. Thereafter, demyelinated slices were incubated in control organotypic slice media, yMSC‐CM and oMSC‐CM for up to 6 days. Fluorescent images show the expression of Neurofilament H (red) as a marker of axons together with the mature oligodendrocyte marker MBP (green) present in the myelin‐like sheaths, as an indicator of myelination at 2 and 6 days *in vitro* (DIV) after lysolecithin‐induced demyelination (a). Scale bar = 100 μm. Quantitative analysis shows the ratio between the area of the slice where NFH colocalizes with MBP and NFH area (b). Note that after 6 DIV only yMSC‐CM enhances *ex vivo* myelin‐like sheath formation while oMSC‐CM leads to similar myelination levels when comparing to control condition. Values are displayed as mean ± *SD*. Experiments are done in triplicate (duplicate for oMSC‐CM). Statistic was performed by two‐way ANOVA and Bonferroni post hoc test. *p* values in the graph title indicate significant difference between the conditions tested. *p < .05

Does the reduced oligodendrogenic activity of oMSCs have a further consequence on CNS regeneration? Remyelination is highly efficient in young individuals, but this efficiency declines with aging (Shields, Gilson, Blakemore, & Franklin, 1999; Sim et al., 2002). Therefore, we next asked whether MSC derived soluble factors could not only increase OPC differentiation but also enhance myelination and, if so whether aging might affect this activity. To address these questions we used a well‐established *ex vivo* myelination model, where organotypic cerebellar slice cultures are shortly exposed to lysolecithin to provoke demyelination (Birgbauer et al., 2004; Jarjour, Zhang, Bauer, Ffrench‐Constant, & Williams, 2012; Zhang, Jarjour, Boyd, & Williams, 2011). For this experiment, yMSC‐CM and oMSC‐CM were prepared exposing MSCs to basic organotypic slice culture medium and the unconditioned medium was used as control. Upon lysolecithin exposure, demyelinated slices were incubated in control medium, yMSC‐CM or oMSC‐CM and were fixed at 2 and 6 days *in vitro* (DIV) post demyelination (Figure 3a). Generation of myelin‐like sheaths around the axons was determined by the ratio between the MBP/NF‐H colocalization area (myelinated axons) and the total NF‐H area (overall axons). This often equates to compact myelin formation in this model (Zhang et al., 2011) (for more details see Supporting Information Movie S1). Slices exposed to yMSC‐CM showed an increase in myelination when compared to control, while there was no difference between control and oMSC‐CM treated slices (Figure 3b). Hence, aging suppresses the ability of MSCs to stimulate the generation of oligodendrocytes and subsequent myelin‐like sheath formation in an *ex vivo* model.

### 3.3 Aging interferes with the capacity of MSCs to enhance OPC differentiation during CNS remyelination *in vivo*

**Figure 4:**
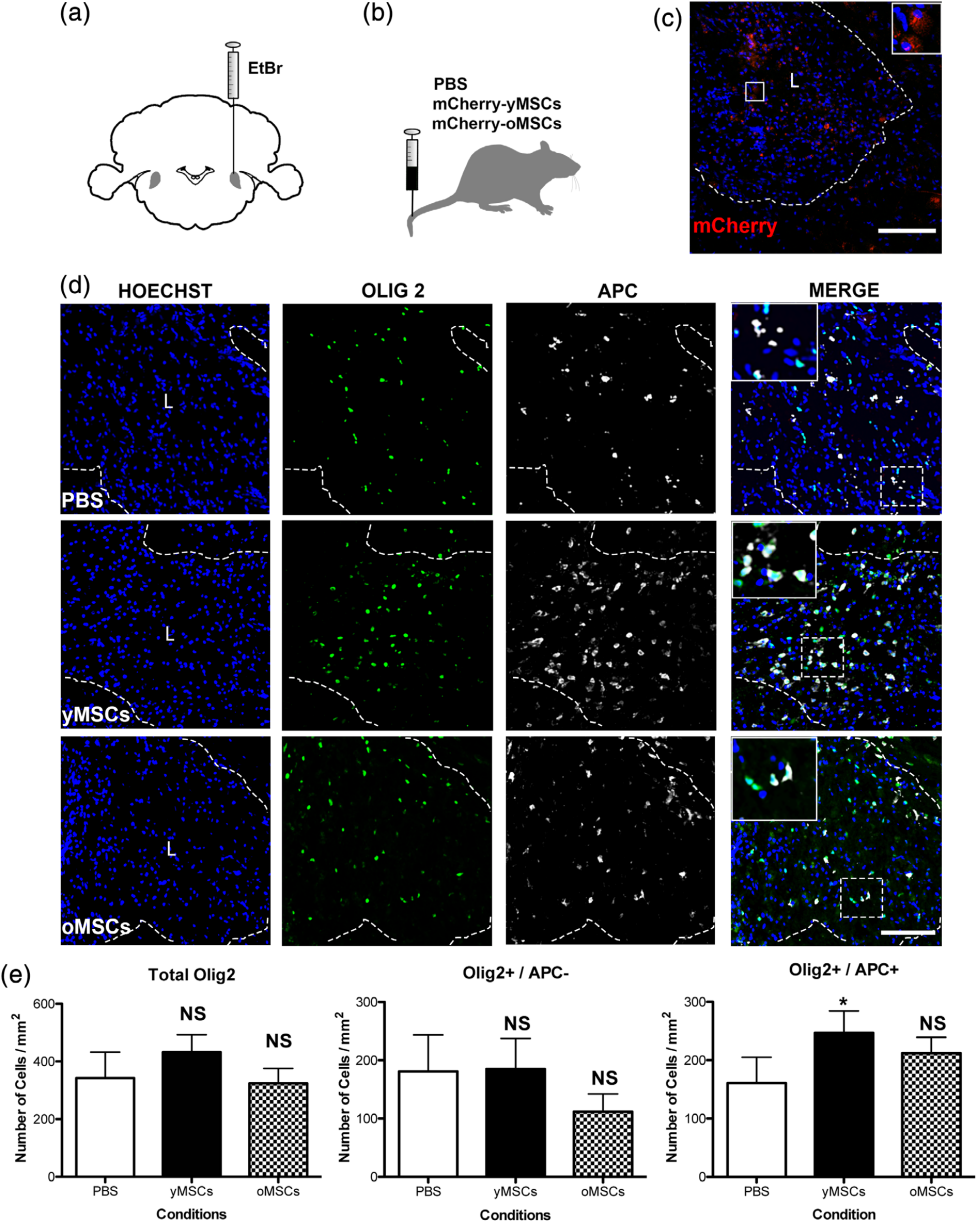
Only transplanted MSCs derived from young donors boost OPC differentiation during CNS remyelination. Twelve months old rats were demyelinated by ethidium bromide (EtBr) injection into the caudal cerebellar peduncle (a). One, two, and three days after demyelination, rats were systemically transplanted with yMSCs or oMSCs that express mCherry for their future detection (b). PBS is used as vehicle control. Fluorescent image shows the presence of mCherry‐expressing MSCs in the demyelinated area at 21 days post‐lesion (dpl) (c). Scale bar = 200 μm. Fluorescent images show the presence of OPCs (Olig2+/APC−) and the generation of new oligodendrocytes (Olig2+/APC+) in the lesion site of the different animal groups (d). Hoechst shows nuclei counterstaining. Dashed lines denote demyelinating lesion area (L). Scale bar = 100 μm. In merge images, the inset shows a magnification of the area delimited by the square. Quantitative analysis shows the number of all oligodendroglial lineage cells (Olig2+), OPCs, and differentiated oligodendrocytes within the demyelinated lesion area (mm^2^) of the distinct animal groups at 21 dpl (e). Note that only the animals that were transplanted with yMSCs display a higher number of differentiated oligodendrocytes (Olig2+/APC+) within the lesion site. Values are displayed as mean ± *SD*. Five animals were analyzed for the PBS group while four animals were analyzed for the group transplanted either with yMSCs or oMSCs. Statistics were performed by one‐way ANOVA and the Tukey post hoc test. *p < .05; NS, not significant

We further explored whether the age‐related restriction in the ability of MSCs to promote the generation of oligodendrocytes and enhance remyelination is also observed in an *in vivo* context. We systemically transplanted, through the tail vein, 4.5–6.0 × 10^6^ mCherry‐expressing yMSCs or oMSCs in adult rats after bilateral demyelination of the caudal cerebellar peduncles (CCP) (Figure 4a,b; Woodruff & Franklin, 1999). PBS intravenous administration was used as a vehicle control. In this model, OPC differentiation occurs very efficiently in young animals, however, it is impaired with increasing age. Rats older than 12 months already display a reduced rate of OPC differentiation contributing to a significant delay in remyelination compared to young animals (<3 months; Shields et al., 1999; Sim et al., 2002). Therefore, middle age rats (12 months old) were used and sacrificed at 21 days post‐lesion (dpl) to assess treatment efficiency.

We used mCherry labeling to evaluate whether systemically transplanted MSCs survive and reach the lesion area and, most likely, exert a paracrine impact on neighboring CNS‐resident cells. Following the mCherry signal, we found that transplanted MSCs were able to reach the demyelinating lesion (Figure 4c). Regardless of the donor's age, we found approximately 65 ± 25 mCherry‐expressing MSCs per 1 mm^2^ within the lesion area. Olig2 is expressed in all cells from the oligodendroglial lineage and we evaluated the expression of adenomatous polyposis coli (APC) to distinguish between OPCs and differentiated oligodendrocytes. No difference was found among all groups in the total number of recruited Olig2+/APC− undifferentiated OPCs; however, only the animals that received yMSCs displayed a higher number of differentiated Olig2+/APC+ oligodendrocytes within the lesion site, while transplanted oMSCs exerted no significant effect on OPC differentiation (Figure 4d,e). During remyelination, activated and proliferating OPCs can be detected by the expression of the transcription factor Nkx2.2 and Ki67, respectively (Watanabe, Hadzic, & Nishiyama, 2004). Regardless of donor's age, transplanted MSCs did not alter the number of Nkx2.2+/Ki67+ proliferating OPCs (Supporting Information Figure S3a,b). These findings together suggest that MSCs increased the number of new oligodendrocytes generated by boosting OPC differentiation instead of indirectly enhancing OPC proliferation. As expected, none of the mCherry positive cells were found to coexpress Olig2 (Supporting Information Figure S4), arguing against transdifferentiation of transplanted MSCs to oligodendrocytes.

**Figure 5:**
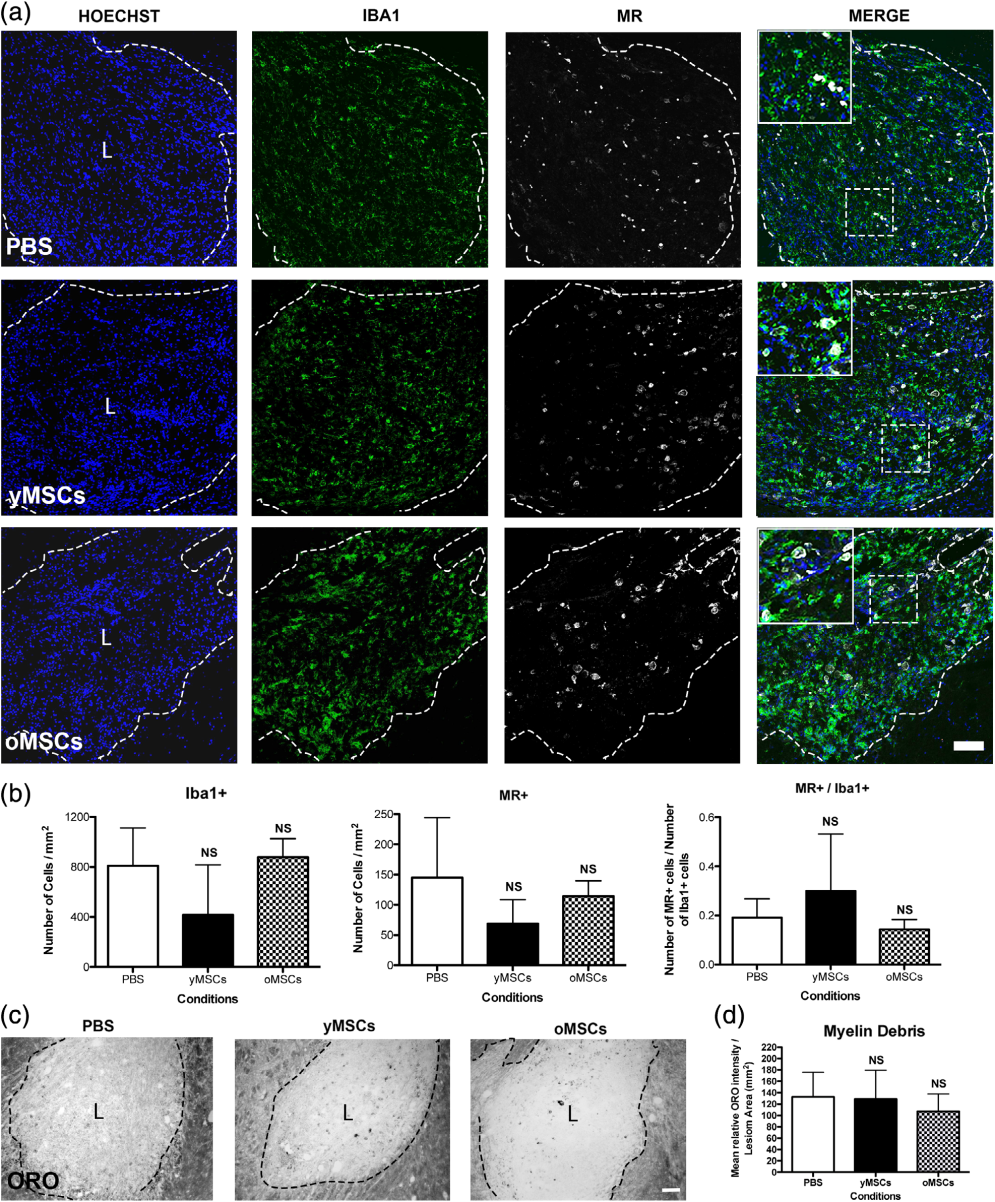
Independently from donors age, transplanted MSCs do not alter macrophage/microglia response during CNS remyelination. Twelve months old rats were demyelinated by ethidium bromide injection into the caudal cerebellar peduncle. One, two, and three days after demyelination, rats were systemically transplanted with mCherry‐expressing yMSCs or oMSCs. PBS is used as vehicle control. Fluorescent images show the lesion site of the different animal groups at 21 dpl, the presence of macrophages/microglia (Iba1+) and their expression of mannose receptor (MR+) evidencing M2 anti‐inflammatory state (a). Hoechst shows nuclei counterstaining. Dashed lines denote demyelinating lesion area (L). Scale bar = 100 μm. In merge images, the inset shows a magnification of the area delimited by the square. Quantitative analysis shows the number of macrophage/microglia cells (Iba1+) and the number of anti‐inflammatory cells (MR+) within the lesion area (mm^2^) as well as the proportion of anti‐inflammatory cells among the total macrophage/microglia cell population (MR+/Iba1+) (b). Note that none of transplanted groups shows significant difference on the macrophage/microglia parameters measured respect to vehicle group. Phase contrast images show oil red O (ORO) staining to detect the presence of myelin debris within the lesion site in the different animal group at 21 dpl (c). Scale bar = 100 μm. Quantification analysis shows the mean relative ORO intensity per lesion area (mm^2^) (d). Note that none of transplanted groups shows a significant difference in the clearance of myelin debris respect to vehicle group. Values are displayed as mean ± *SD*. Five animals were analyzed for the PBS group while four animals were analyzed for the group transplanted either with yMSCs or oMSCs. Statistics were performed by one‐way ANOVA and the Tukey post hoc test. NS, not significant

During remyelination, OPC differentiation is favored by anti‐inflammatory (M2) macrophages/microglia and inhibited by the presence of myelin debris (Miron et al., 2013). Therefore, we evaluated whether transplanted MSCs alter these particular properties that might indirectly influence OPC differentiation. Regardless of the donor's age, transplanted MSCs neither affect the number of Iba1+ macrophages/microglia present within the demyelinated lesion nor their M2 polarization revealed by the appearance of mannose receptor (MR)‐expressing subpopulation (Figure 5a,b). In addition, Oil Red O stainings, widely used to detect myelin debris in the lesion area (for example in Ruckh et al. [2012]), revealed no effect on the clearance of myelin debris after MSCs treatments (Figure 5c,d). These data together with the previous *in vitro* findings suggest that transplanted MSCs directly exert their action on CNS‐resident progenitor cells rather than modulating macrophages/microglia function and indirectly influencing OPCs differentiation.

**Figure 6:**
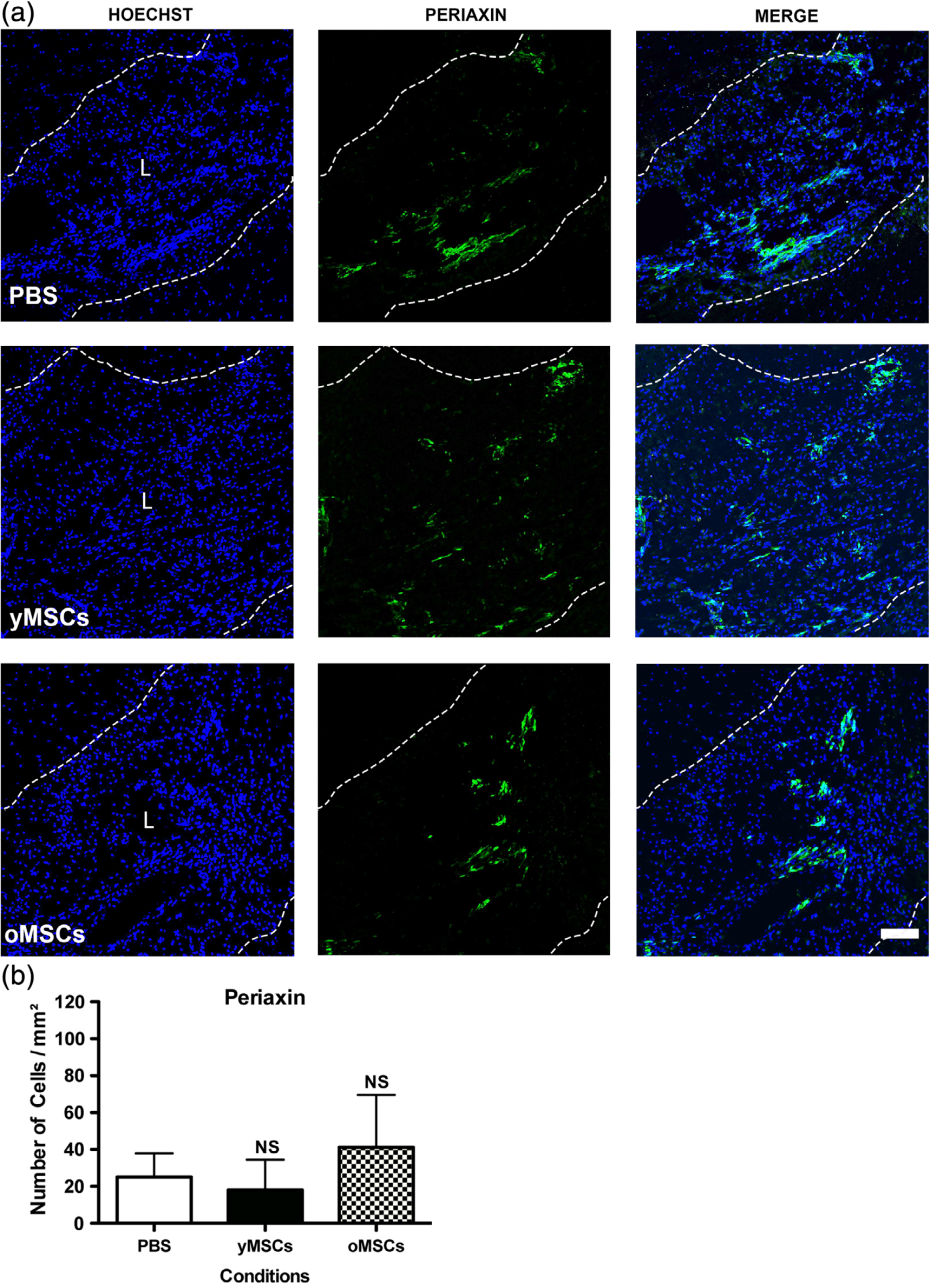
Independently from donors age, transplanted MSCs do not alter the presence of Schwann cells during CNS remyelination. Twelve months old rats were demyelinated by ethidium bromide injection into the caudal cerebellar peduncle. One, two, and three days after demyelination, rats were systemically transplanted with mCherry‐expressing yMSCs or oMSCs. PBS is used as vehicle control. Fluorescent images show the lesion site of the different animal groups at 21 dpl and the presence of Schwann cells (a). Scale bar = 100 μm. Hoechst shows nuclei counterstaining. Dashed lines denote demyelinating lesion area (L). Quantitative analysis shows the number of Periaxin‐expressing cells present within the lesion area (b). Note that none of transplanted groups shows significant difference in the number of Schwann cells present within the lesion respect to vehicle group. Values are displayed as mean ± *SD*. Five animals were analyzed for the PBS group while four animals were analyzed for the group transplanted either with yMSCs or oMSCs. Statistics were performed by one‐way ANOVA and the Tukey post hoc test. NS, not significant

Finally, as OPC derived Schwann cells can also contribute to remyelination in the CNS (Zawadzka et al., 2010), we evaluated whether transplanted MSCs influence this particular OPC feature. No changes were found in the number of Periaxin‐positive cells, a well‐established Schwann cell marker (Zawadzka et al., 2010), in response to transplantation, indicating that MSCs do not alter OPC differentiation toward Schwann cells (Figure 6a,b). No cross‐reaction between Periaxin and the inflammation marker Iba1 was observed (Supporting Information Figure S5), supporting the specificity of Periaxin to label Schwann cells. In summary, consistent with the previous *in vitro* and *ex vivo* findings, aging suppresses the capacity of transplanted MSCs to directly boost OPC differentiation and the further generation of new oligodendrocytes during *in vivo* remyelination.

## 4 DISCUSSION

Aging affects homeostasis and reduces the regenerative capacity of various tissues and organs (Alt et al., 2012; Fulle et al., 2012; Ruckh et al., 2012). Besides systemic and local environmental cues, restrictions in the tissue‐resident adult stem cells and their properties contribute to the age‐related reduction in the regenerative potential. Here, we have shown that soluble factors derived from aged MSCs have a reduced ability to promote oligodendrogenesis in CNS stem and progenitor cells. It has been shown that aging influences the neurotrophic activity of MSCs (Brohlin, Kingham, Novikova, Novikov, & Wiberg, 2012), therefore it is likely that aged MSCs display a lower capacity to synthesize and/or release their yet unknown “oligodendrocyte differentiation factor(s)” when compared with MSCs derived from young donors. Despite the presence of an oligodendrogenic activity, aged MSCs failed to boost OPC differentiation during remyelination. Moreover, while yMSC were able to enhance the generation of myelin‐like sheaths in an *ex vivo* model, oMSC lacked this capacity, suggesting that even if oMSCs are able to promote OPC differentiation *in vitro* their effect may be limited during *in vivo* remyelination.

Aging decreases OPC differentiation and impairs endogenous CNS remyelination (Franklin & Ffrench‐Constant, 2008; Shen et al., 2008; Shields et al., 1999; Sim et al., 2002). Nevertheless, in a previous study, it has been shown that remyelination efficiency in old animals can be restored after exposure to a youthful systemic milieu via heterochronic parabiosis (Ruckh et al., 2012) or treatment with molecules such as 9‐cis retinoic acid (Huang et al., 2011). Together, these observations suggest that age‐related myelin regeneration restrictions can be overcome by the contribution of a strong regenerative microenvironment or stimulus. Here, we observed an enhancement in OPC differentiation during remyelination in middle‐aged CNS in response to systemic transplantation of MSCs obtained from young donors. Interestingly, even though only a few transplanted cells reach the lesion area, this pro‐regenerative effect exerted by MSCs might be supported by this fact that enables a closer interaction with CNS‐resident OPCs. These data suggest that transplanted MSCs might change the systemic and/or the CNS milieu exerting their oligodendrogenic activity and overcoming intrinsic age‐related myelin repair limitations. However, similar to *ex vivo* myelination, aging abolishes this MSC pro‐regenerative capacity as transplanted aged MSCs fail to boost OPC differentiation in the CNS of middle age recipients.

The age of 17–20 months for oMSCs was chosen for the following reasons: (a) 17–20 months reflects approximately a human age that ranges between 50 and 60 years, given the mean longevity of male Fischer 344 rats of 642 days (Chesky & Rockstein, 1976) and the mean life expectancy of the human male population of 69 years (“World Health Statistics 2016: Monitoring Health for the SDGs”. World Health Organization); (b) life expectancy of MS patients is 10–15 years lower than that of the unaffected population (Compston & Coles, 2008), hence 55–60 years. Thus, we assume that 17–20 month old rats have a chronological/biological age of later stage MS patients, which are currently in the scope of MSC‐MS trials.

Since MSCs display immunomodulatory and neuroprotective activities, systemic autologous MSC therapy has been proposed for the treatment of MS (Connick et al., 2012; Freedman et al., 2010). Here, we have shown for the first time that soluble factors derived from MSCs enhance *ex vivo* myelin‐like sheath formation and that systemic MSCs transplantation boosts the generation of oligodendrocytes during CNS remyelination. Thus, this study strengthens the pro‐regenerative activity of MSCs and provides a further rationale for autologous MSC transplantation in MS therapy. In addition, and as an alternative strategy, a continuous pharmacological supply of the MSC‐derived oligodendrogenic factor(s), which still need to be identified, could have a controlled and specific effect in the generation of new oligodendrocytes enhancing remyelination. However, this study argues on caution when developing this therapy for older MS patients as aging suppresses MSCs pro‐oligodendrogenic activity. Thus, it clearly urges for a careful revision when designing clinical MSC‐based trials for MS treatment. The use of young‐donor derived MSCs, for example, umbilical cord‐derived MSCs, might be more efficient than autologous MSCs from elderly patients.

## CONFLICT OF INTEREST

The authors declare that there are no conflicts of interest.

5

## Supporting information


**Movie S1** Confocal video showing the Z stacks of demyelinated cerebellar slices treated with yMSC‐CM for 8 days. Myelin (MBP) is shown in red and axons (NFH) is shown in green. The video shows in detail how the myelin is ensheathing the axons.

**Figure S1** Regardless of the donors age, conditioned media derived from MSCs with different passages display a similar oligodendrogenic activity. Growth curve comparison between increasing passage numbers in yMSCs (a) as well in oMSCs (b). Note that long‐term *in vitro* expansion influences MSCs growth rate. Quantification of bioluminescence data shows MBP promoter activation (as luciferase activity) in NSCs either under control conditions or exposed to conditioned medium from yMSCs (c) and oMSCs (d) from increasing passage numbers. Note that independently of the passage number, conditioned medium derived from MSCs similarly increase MBP promoter activity. Values are displayed as mean ± *SD*. Experiments were performed in triplicate and two‐way ANOVA was used for statistical analysis. The *p* values given in graph title indicate significant difference between the increasing MSCs passages (a and b). For bioluminescence assays, experiments were performed in triplicate and one‐way ANOVA‐Tukey post hoc was used for statistical analysis. *Differences between passage 2 and passage 6; ^#^differences between passage 2 and passage 4. **o ^##^ *p* < .01; *** o ^###^ *p* < .001.

**Figure S2** Soluble factors derived from yMSCs do not affect cell proliferation and survival during OPC differentiation. Differentiating OPCs were exposed to yMSC‐CM and the proportion of proliferating and apoptotic cells was determined. At 0, 2, 4, and 6 days *in vitro* the percentage of Ki67+ (a) and Caspase 3+ (b) cells were determined to evaluate cell proliferation and survival, respectively. No differences were observed in OPCs exposed to yMSC‐CM compared cells incubated under control conditions. NS, not significant.

**Figure S3** Transplanted MSCs do not affect OPC activation and proliferation during CNS remyelination. Twelve months old rats were demyelinated by ethidium bromide injection into the caudal cerebellar peduncle. One, two, and three days after demyelination, yMSCs or oMSCs were systemically transplanted. PBS is used as vehicle control. Quantitative analysis shows the number of proliferating cells (Ki67+) (a) and the number of activated proliferating OPCs (Nkx2.2+/Ki67) (b) within the lesion area (mm^2^) at 21 dpl. Note that none of transplanted groups shows significant difference in the number of proliferating cells and proliferating OPCs respect to the vehicle group. NS, not significant.

**Figure S4** Transplanted MSCs do not transdifferentiate into cells from the oligodendroglial lineage during CNS remyelination. Twelve months old rats were demyelinated by ethidium bromide injection into the caudal cerebellar peduncle. One, two, and three days after demyelination, rats were systemically transplanted with yMSCs that express mCherry for their detection. Images show the presence of mCherry‐expressing MSCs (red) as well as Olig2+ cells (green) at 21 dpl. Hoechst shows nuclei counterstaining (blue). Dashed lines denote demyelinating lesion area (L). Scale bar = 50 μm. Inset in merge image shows a high magnification of the area limited by the square. Note that none of the transplanted mCherry‐expressing MSCs coexpress the oligodendrocyte lineage marker Olig2.

**Figure S5** Periaxin‐positive Schwann Cells do not coexpress the inflammatory cell marker Iba1 during CNS remyelination. Twelve months old rats were demyelinated by ethidium bromide injection into the caudal cerebellar peduncle. Left panel shows the presence of Periaxin‐expressing Schwann cells (green) and Iba1+ positive inflammatory cells (red) at 21 dpl. Hoechst shows nuclei counterstaining (blue). Dashed lines denote demyelinating lesion area (L). Scale bar = 100 μm. Rest of the panels show a higher magnification of the area limited by the square. In merge image, the inset shows a magnification of the area delimited by the square. Note the absence of Periaxin‐positive Schwann cells coexpressing the inflammatory cell marker Iba1.

## Data Availability

The data that support the findings of this study are available from the corresponding author upon reasonable request.
