# Supplementary figures and images for: Aging restricts the ability of mesenchymal stem cells to promote the generation of oligodendrocytes during remyelination
Source: Glia. 2019 Apr 30;67(8):1510–25. doi: 10.1002/glia.23624 (PMC6618006; doi:10.1002/glia.23624)

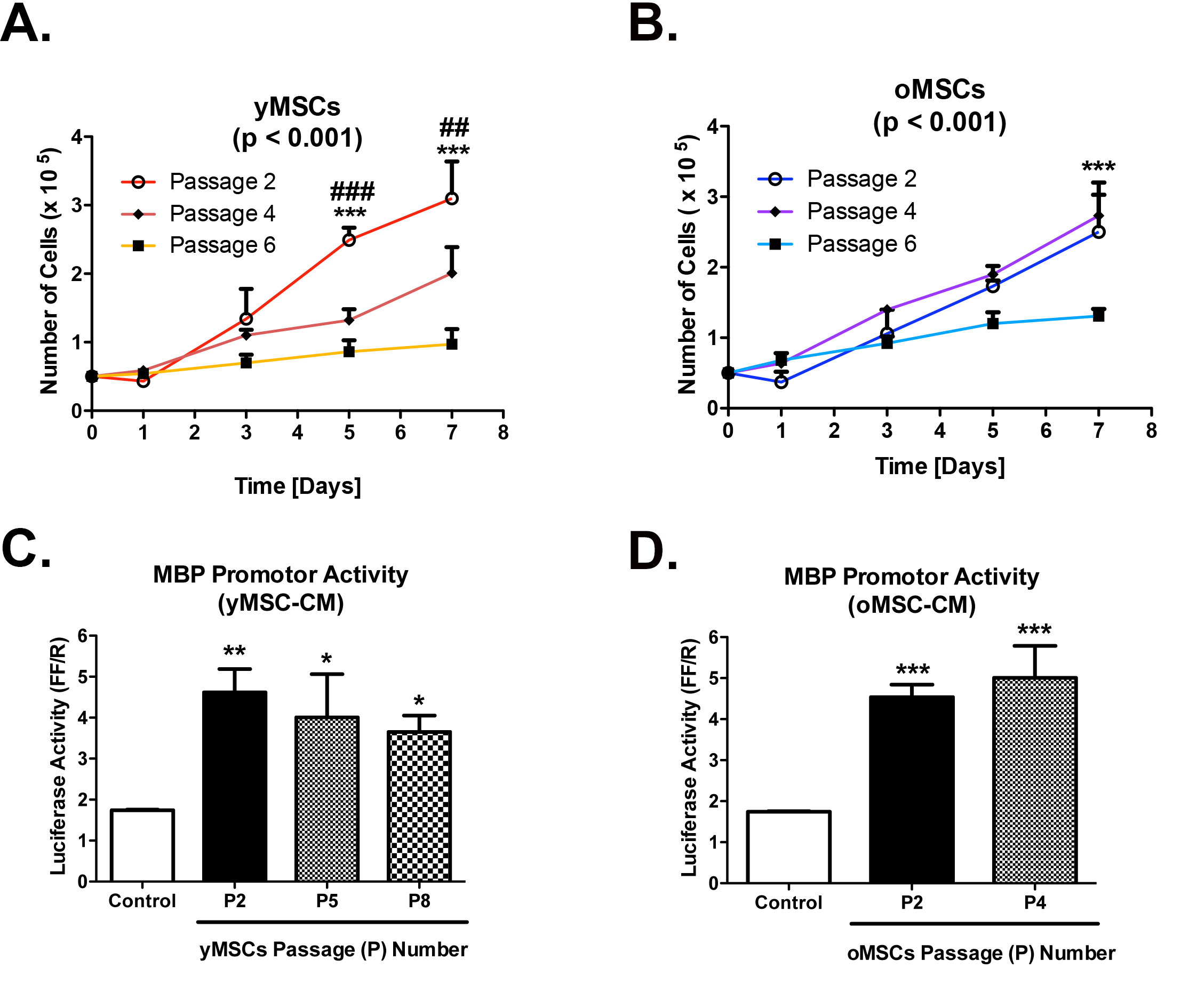

Supplement: Supplementary file 2 — Figure S1 Regardless of the donors age, conditioned media derived from MSCs with different passages display a similar oligodendrogenic activity. Growth curve comparison between increasing passage numbers in yMSCs (a) as well in oMSCs (b). Note that long‐term in vitro expansion influences MSCs growth rate. Quantification of bioluminescence data shows MBP promoter activation (as luciferase activity) in NSCs either under control conditions or exposed to conditioned medium from yMSCs (c) and oMSCs (d) from increasing passage numbers. Note that independently of the passage number, conditioned medium derived from MSCs similarly increase MBP promoter activity. Values are displayed as mean ± SD. Experiments were performed in triplicate and two‐way ANOVA was used for statistical analysis. The p values given in graph title indicate significant difference between the increasing MSCs passages (a and b). For bioluminescence assays, experiments were performed in triplicate and one‐way ANOVA‐Tukey post hoc was used for statistical analysis. *Differences between passage 2 and passage 6; #differences between passage 2 and passage 4. **o ## p < .01; *** o ### p < .001. [file GLIA-67-1510-s002.tif]

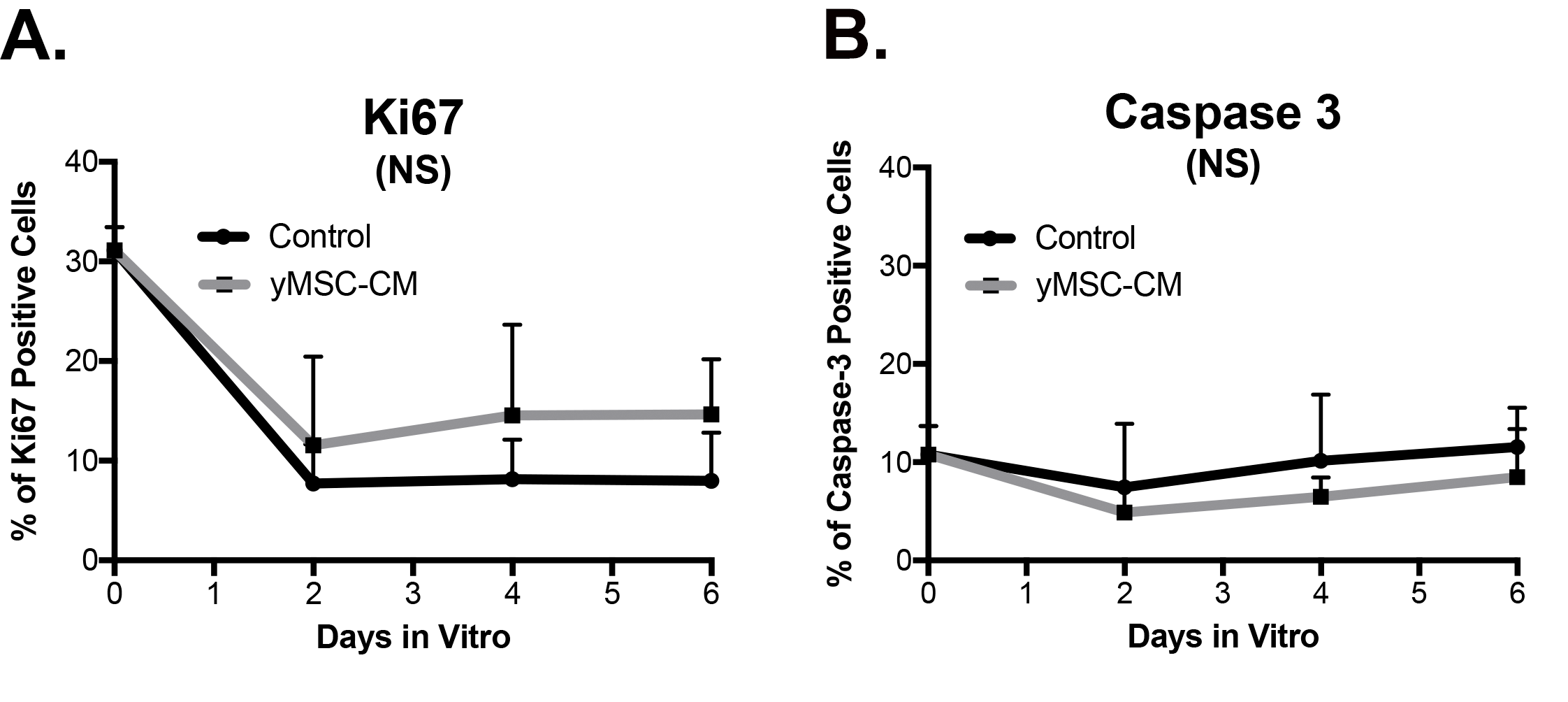

Supplement: Supplementary file 3 — Figure S2 Soluble factors derived from yMSCs do not affect cell proliferation and survival during OPC differentiation. Differentiating OPCs were exposed to yMSC‐CM and the proportion of proliferating and apoptotic cells was determined. At 0, 2, 4, and 6 days in vitro the percentage of Ki67+ (a) and Caspase 3+ (b) cells were determined to evaluate cell proliferation and survival, respectively. No differences were observed in OPCs exposed to yMSC‐CM compared cells incubated under control conditions. NS, not significant. [file GLIA-67-1510-s003.tif]

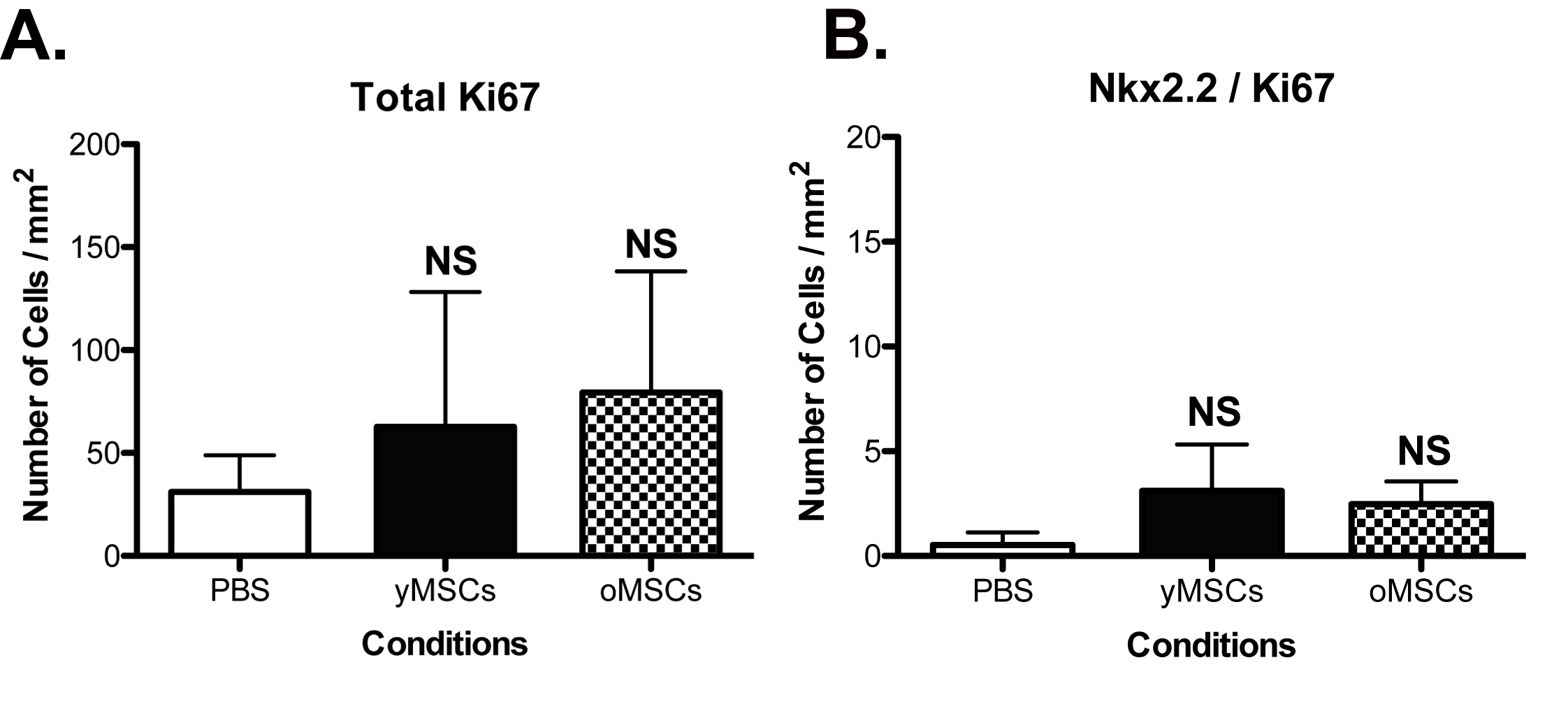

Supplement: Supplementary file 4 — Figure S3 Transplanted MSCs do not affect OPC activation and proliferation during CNS remyelination. Twelve months old rats were demyelinated by ethidium bromide injection into the caudal cerebellar peduncle. One, two, and three days after demyelination, yMSCs or oMSCs were systemically transplanted. PBS is used as vehicle control. Quantitative analysis shows the number of proliferating cells (Ki67+) (a) and the number of activated proliferating OPCs (Nkx2.2+/Ki67) (b) within the lesion area (mm2) at 21 dpl. Note that none of transplanted groups shows significant difference in the number of proliferating cells and proliferating OPCs respect to the vehicle group. NS, not significant. [file GLIA-67-1510-s004.tif]

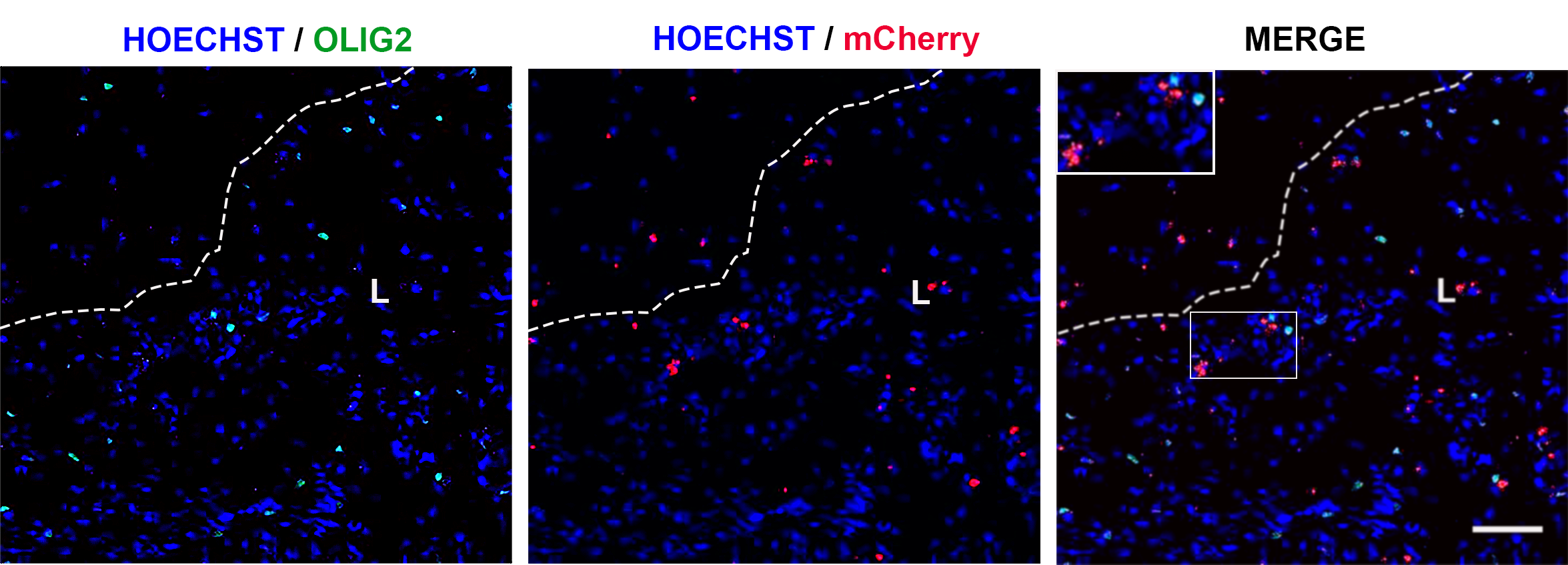

Supplement: Supplementary file 5 — Figure S4 Transplanted MSCs do not transdifferentiate into cells from the oligodendroglial lineage during CNS remyelination. Twelve months old rats were demyelinated by ethidium bromide injection into the caudal cerebellar peduncle. One, two, and three days after demyelination, rats were systemically transplanted with yMSCs that express mCherry for their detection. Images show the presence of mCherry‐expressing MSCs (red) as well as Olig2+ cells (green) at 21 dpl. Hoechst shows nuclei counterstaining (blue). Dashed lines denote demyelinating lesion area (L). Scale bar = 50 μm. Inset in merge image shows a high magnification of the area limited by the square. Note that none of the transplanted mCherry‐expressing MSCs coexpress the oligodendrocyte lineage marker Olig2. [file GLIA-67-1510-s005.tif]

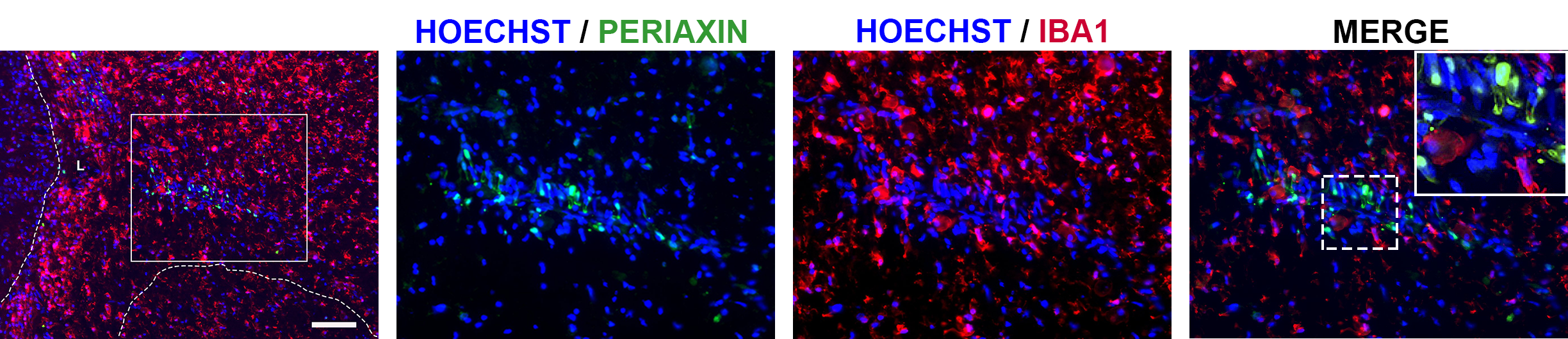

Supplement: Supplementary file 6 — Figure S5 Periaxin‐positive Schwann Cells do not coexpress the inflammatory cell marker Iba1 during CNS remyelination. Twelve months old rats were demyelinated by ethidium bromide injection into the caudal cerebellar peduncle. Left panel shows the presence of Periaxin‐expressing Schwann cells (green) and Iba1+ positive inflammatory cells (red) at 21 dpl. Hoechst shows nuclei counterstaining (blue). Dashed lines denote demyelinating lesion area (L). Scale bar = 100 μm. Rest of the panels show a higher magnification of the area limited by the square. In merge image, the inset shows a magnification of the area delimited by the square. Note the absence of Periaxin‐positive Schwann cells coexpressing the inflammatory cell marker Iba1. [file GLIA-67-1510-s006.tif]
